# Habitual patellar dislocation in extension in children with immature epiphysis: a report of three cases

**DOI:** 10.1186/s12891-026-09620-4

**Published:** 2026-03-23

**Authors:** Zhibin Yu, Jun Zhang, Changhui Shen, Yuhao Zhang, Chaoran Wang, Daohong Zhao, Yuqi Li, Yushan Wan

**Affiliations:** Department of Orthopaedics, Second Affiliated Hospital of Kunming Medical University, Kunming Medical University, Kunming, Yunnan 650000 China

**Keywords:** Habitual patellar dislocation in extension, Children, Patent epiphysis, Patella alta, Trochlear dysplasia, Treatment

## Abstract

**Background:**

Pediatric habitual patellar dislocation in extension (E-HPD) is a rare and specific form of patellar dislocation with limited reports in the literature. For children with immature epiphyses, the gold standard for E-HPD surgery has not been established, and the appropriate treatment is controversial. We report three cases of pediatric E-HPD with epiphyseal immaturity and explore the etiology, risk factors, and treatment of E-HPD in children.

**Methods:**

This article describes three cases of children with E-HPD whose epiphyses were not closed because of skeletal immaturity. The children ranged in age from 7 to 11 years, and all were female. Two patients underwent a combination of medial patellofemoral ligament reconstruction and lateral retinacular release, and one chose to continue conservative treatment. Clinical outcomes were assessed using the Kujala, International Knee Documentation Committee (IKDC), Lysholm, and visual analog scale (VAS) scores and presence of patellar instability, and radiographic data were collected from medical records.

**Results:**

One patient chose to continue conservative treatment, but the patellar dislocation did not improve. The other two patients were treated with medial patellofemoral ligament reconstruction and lateral retinacular release. Both patients had good outcomes with no recurrent dislocations during their respective 14- and 29-month follow-ups. All postoperative scores (Kujala, IKDC, Lysholm, and VAS for pain) significantly improved compared to preoperative levels.

## Introduction

Patellar dislocation has a complex etiology and many classifications. The most common clinical classification of patellar dislocations is habitual and recurrent dislocations. Habitual patellar dislocation is patellar movement in the lateral direction out of the trochlea during each knee flexion and extension exercise cycle, and most studies define it as patellar dislocation during flexion and reduction during knee extension [1–4]. Extension-type habitual patellar dislocation (habitual patellar dislocation in extension, E-HPD) is a rare type of habitual patellar dislocation that is characterized by dislocation of the patella on extension or at the start of flexion of the knee [5]. This type of patellar dislocation is occasionally observed in pediatric patients but has rarely been reported in the literature.

Children’s epiphyses are not closed, and the manipulation of bones during surgery can affect bone development; therefore, these factors should be considered before any surgical intervention. For pediatric patients, most surgeons choose medial patellofemoral ligament (MPFL) reconstruction to treat patellar dislocation, and soft tissue surgeries, such as the release of the lateral retinaculum and quadricepsplasty, are chosen according to the patient’s condition, usually with a good prognosis. E-HPD is more common in children and is often associated with skeletal dysplasia or deformity. Therefore, choosing an appropriate surgical method and determining its efficacy without advocating the use of tibial tuberosity displacement, trochleoplasty, tibial osteotomy, femoral osteotomy, patellar osteotomy, or other bony surgeries is challenging. E-HPD is rare, with few reports in the literature and even fewer reports on the surgical treatment of E-HPD in childhood. The diagnostic and treatment details of three patients with extension-type patellar dislocation in our department are reported (Table 1).

**Table 1:** Basic information and imaging parameters of patients

Patient	Age (years)	Sex	Side off	Caton-Deschamps index	IS ratio	TT-TG value	Femoral trochlear groove angle	Femoral anteversion	Dejour classification
1	7.3	Female	Right	1.31	1.47	14.0	152.7°	-	Type D
2	11.2	Female	Left	1.36	1.38	17.00	151.7°	21.0°	Type C
3	11.6	Female	Left	-	-	20.20	148.2°	-	Type C
Average	10.0			1.33	1.43	14.03	150.9°	21.0°	

Missing values are attributable to the unavailability of certain original imaging data

*IS ratio* Insall-Salvati ratio, *TT-TG value* distance between the tibial tubercle and trochlear groove

### Clinical data

#### Case 1

**Fig. 1:**
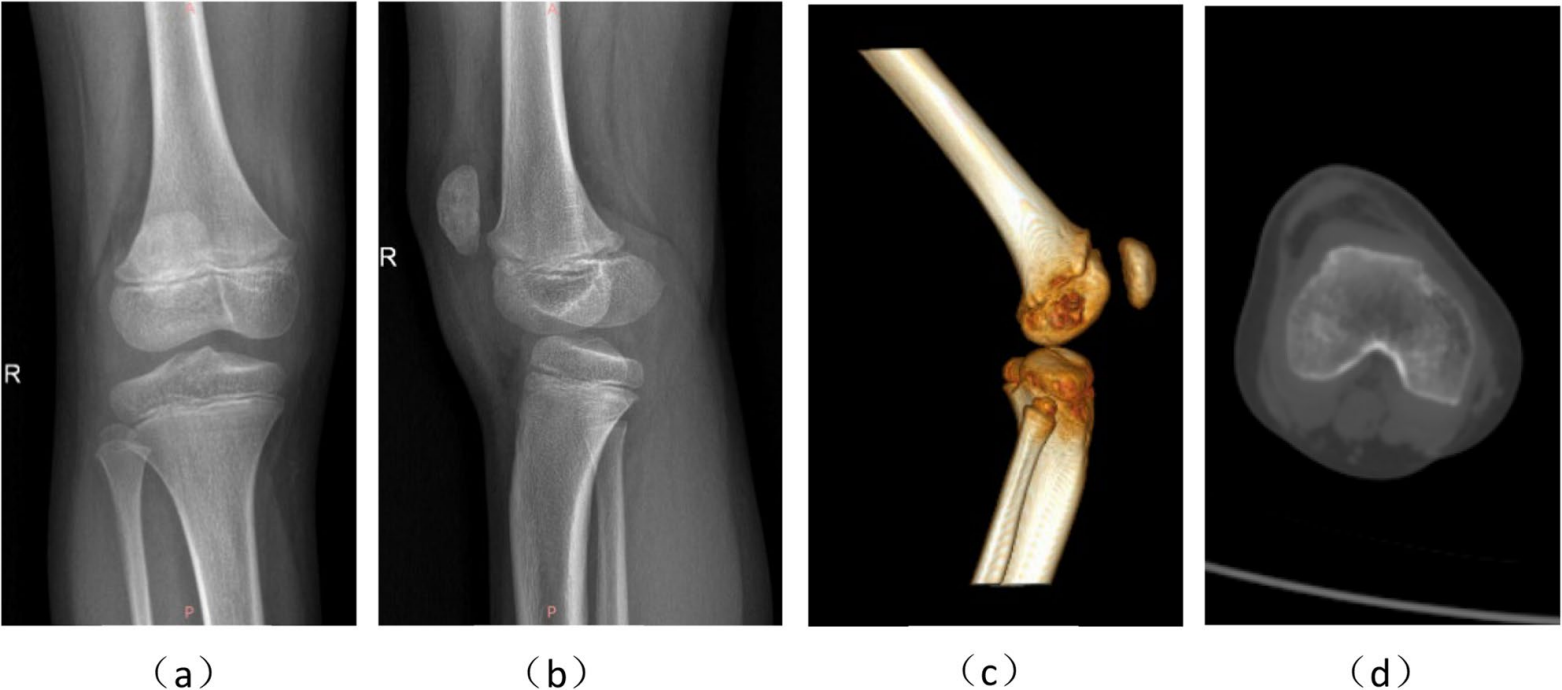
Preoperative radiographs and computed tomography (CT) images of the knee joint. **a** The anteroposterior radiograph of the right knee joint shows a visible, unclosed epiphysis and subluxated patella; (b) lateral radiograph of the right knee joint demonstrating patella alta; **c** CT three-dimensional CT reconstruction of the right knee joint demonstrating patella alta; **d** horizontal CT of the right knee joint showing a low, flat femoral trochlea and the Dejour type D trochlea

 A 7.3-year-old female had experienced repeated dislocation of the patella of the right knee joint for > 3 months without obvious induction. Prolapse occurred often when the knee joint was straightened and could be reset during knee flexion, accompanied by knee joint instability. When squatting and standing, the patient experienced weakness in the right lower limb. After prolonged walking, she experienced weakness in the lower right leg, with slight pain in the knee joint. Physical examination revealed lateral dislocation of the patella when the right knee joint turned from flexion to extension by approximately 5°. Flexion of 30° repositioned the patella, with the patella pushed inward by one degree and tightness of the lateral retinaculum. No obvious pain or movement limitations were observed. Anteroposterior and lateral radiographs of the knee indicated an Insall-Salvati ratio of patellar height and Caton index of 1.47 and 1.31, respectively, suggesting patella alta. The patella was subluxated when the right knee was extended. Computed tomography (CT) images at 0° and 40° of right knee flexion showed trochlear dysplasia with a femoral trochlear groove angle of 152.7°. The Dejour trochlear dysplasia was classified as type D, and the distance between the tibial tubercle and trochlear groove (TT-TG) was 14.0 mm (Fig. 1). The combination of symptoms and auxiliary examinations led to a diagnosis of right E-HPD. Considering both the young age of the patient and the patent epiphysis, lateral knee retinaculum release and MPFL reconstruction was chosen.

**Fig. 2:**
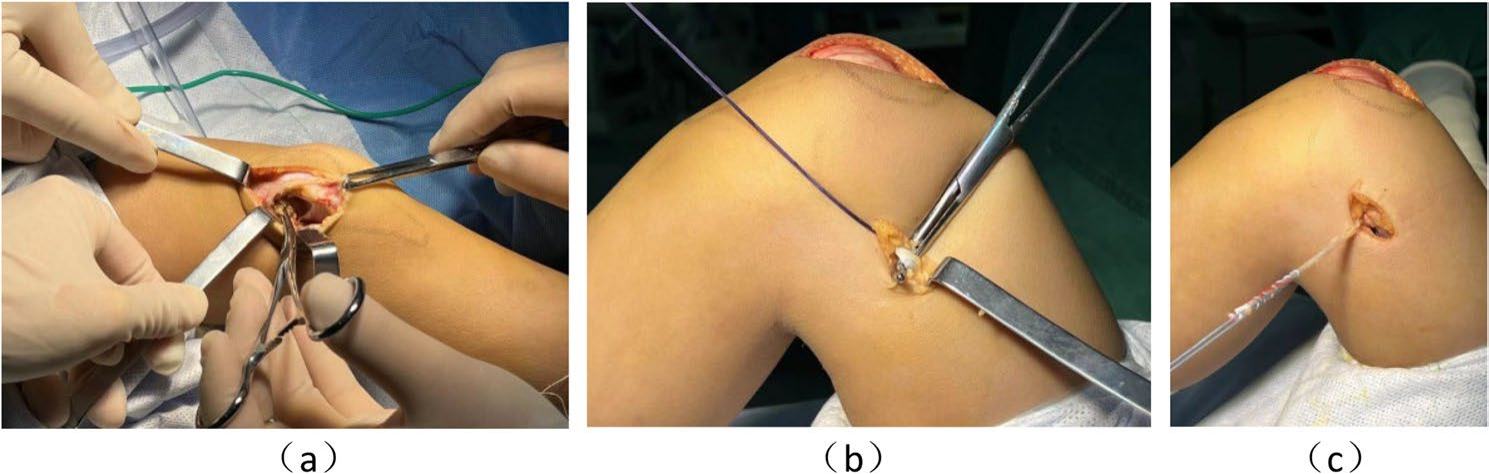
Intraoperative photographs. **a** An incision is made at the center of the anterior aspect of the right knee joint, and the lateral support of the patella is widely released. **b** The adductor magnus tendon is separated. **c** The distal insertion attachment of the adductor magnus tendon is preserved, and the tendon is woven

The patient was administered general anesthesia, and the affected knee joint was flexed and extended preoperatively, to observe the patellar trajectory. Under anesthesia, patellar dislocation could not be induced by flexion or extension of the knee joint. At the beginning of the operation, a 10 cm anterior midline incision was made from the distal femur to the upper part of the tibial tubercle, and the medial and lateral patellar retinaculum and patellar tendon were superficially exposed. The lateral retinaculum was released to relieve adhesions between the lateral retinaculum, subcutaneous tissue, and synovium. A 3 cm longitudinal incision was then made between the adductor magnus tendon and adductor tubercle of the femur. The adductor magnus tendon was separated and dissociated proximally, with a cut at the transition between the adductor magnus tendon and muscle belly and retention of the distal femoral insertion of the adductor magnus tendon, to prepare and weave the tendon (Fig. 2). The graft was finally fixed with two rivets in the middle and upper third of the medial aspect of the patella at the origin of the anatomical MPFL and tightened at 30° of knee flexion.

Postoperatively, the patient was informed of early muscle strength training of the lower limbs, including ankle pump and straight leg-raising exercises. Surgical sutures were removed at two weeks postoperatively. Concurrently, the initial fiberglass cast was replaced with a hinged knee brace, facilitating the commencement of rehabilitation exercises with a controlled range of motion. The patient began both active and passive exercises of the knee joint and quadriceps and gradual weight-bearing under tolerance. The initial angle of activity was set to 0–30°. Four weeks postoperatively, the knee flexion angle reached 90°, and full weight-bearing was allowed; the brace was used for 6–8 weeks. During the 14-month follow-up period, no patellar dislocation recurrence was noted. The Kujala, IKDC, and Lysholm scores improved from 79.0 to 91.0, 70.1 to 87.4, and 70.0 to 86.0, respectively. Visual analog scale (VAS) scores improved from 3.0 points preoperatively to 1.0 point postoperatively.

#### Case 2

**Fig. 3:**
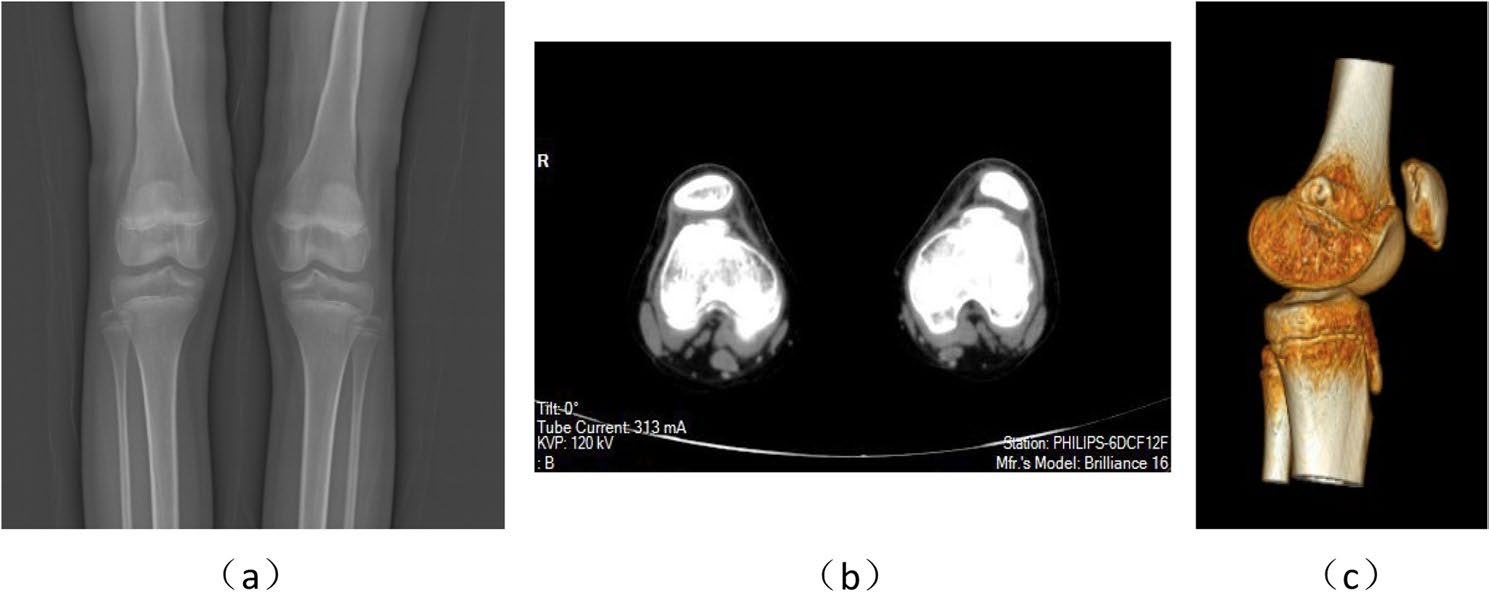
Preoperative radiograph and computed tomography (CT) images of the knee joint in case 2. **a** The anteroposterior radiograph of the bilateral knee joints shows the unclosed epiphysis and left subluxated patella; **b** The horizontal CT image of the bilateral knee joints shows Dejour type C trochlear dysplasia and that the bilateral patella is not in the trochlear groove; **c** The three-dimensional reconstruction of the right knee joint demonstrates patella alta

An 11.2-year-old female had experienced repeated outward prolapse of the patella of the left knee joint since birth. Frequent prolapse occurred when the left knee joint straightened and could be automatically reset with postural changes and knee bending. Frequent lower limb weakness occurred while walking, with knee joint instability, difficulty squatting and standing, and the need for support to stand after squatting. After extended periods of walking, she experienced knee pain and was unable to perform regular sports activities. During physical examination, the patient’s left knee joint dislocated laterally when it changed from flexion to extension by approximately 10°. When slightly flexed to 20°, the knee joint automatically returned to its original position. The patella was pushed inward by 1°. During knee extension and flexion, no obvious pain was reported in the left knee, and no limitation in lower limb movement was observed. Radiographic data of the left knee joint indicated an Insall-Salvati ratio of patellar height and Caton index of 1.38 and 1.36, respectively, suggesting patella alta, and the left knee joint was subluxated compared to the right knee joint during extension. The 0° and 40° CT of the right knee showed trochlear dysplasia with a femoral trochlear groove angle of 151.7°. The Dejour trochlear dysplasia was classified as type C, and the TT-TG measurement was 17.0 mm (Fig. 3).

**Fig. 4:**
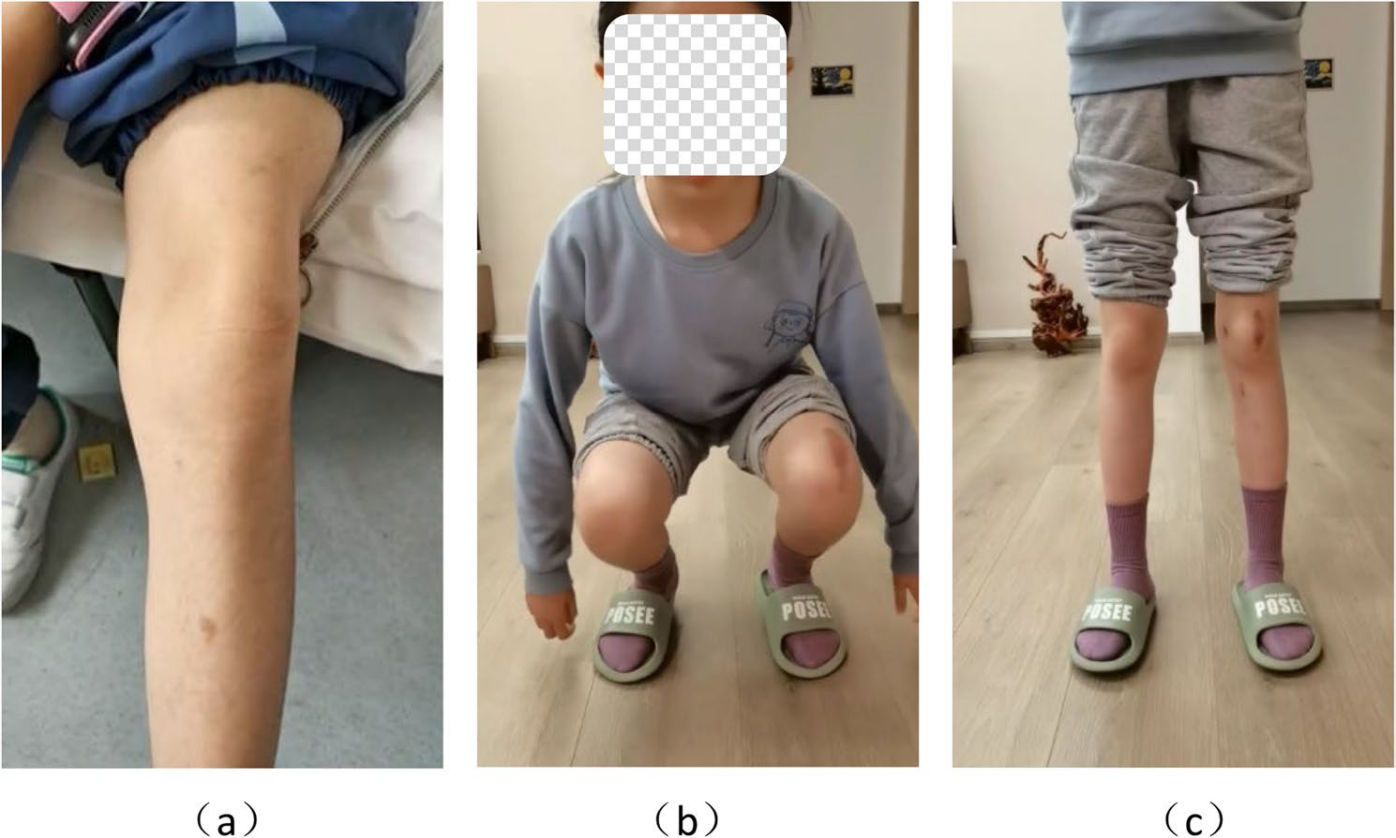
Pre- and postoperative images of patient 2: **a** preoperative appearance of the left knee joint in extension; the patella is visible lateral to the knee joint; **b**, **c** postoperative appearance after 10 months; the left knee joint has a normal range of motion, the patient can squat and stand independently, the patellar track is good, and no dislocation is observed

Based on the patient’s symptoms and auxiliary examinations, the patient was diagnosed with left E-HPD, and surgical intervention and postoperative rehabilitation of the left knee were performed in the same manner as described in the first case. Ten months post-surgery, the left knee joint functions significantly improved compared to the preoperative functions, with good patellar trajectory during movement (Fig. 4) and no pain or other discomfort. During the 29-month follow-up period, no recurrence of patellar dislocation was observed. The Kujala, IKDC, and Lysholm scores improved from 84.0 to 94.0, 66.7 to 80.5, and 71 to 90, respectively. The VAS score improved from 5.0 to 2.0.

#### Case 3

**Fig. 5:**
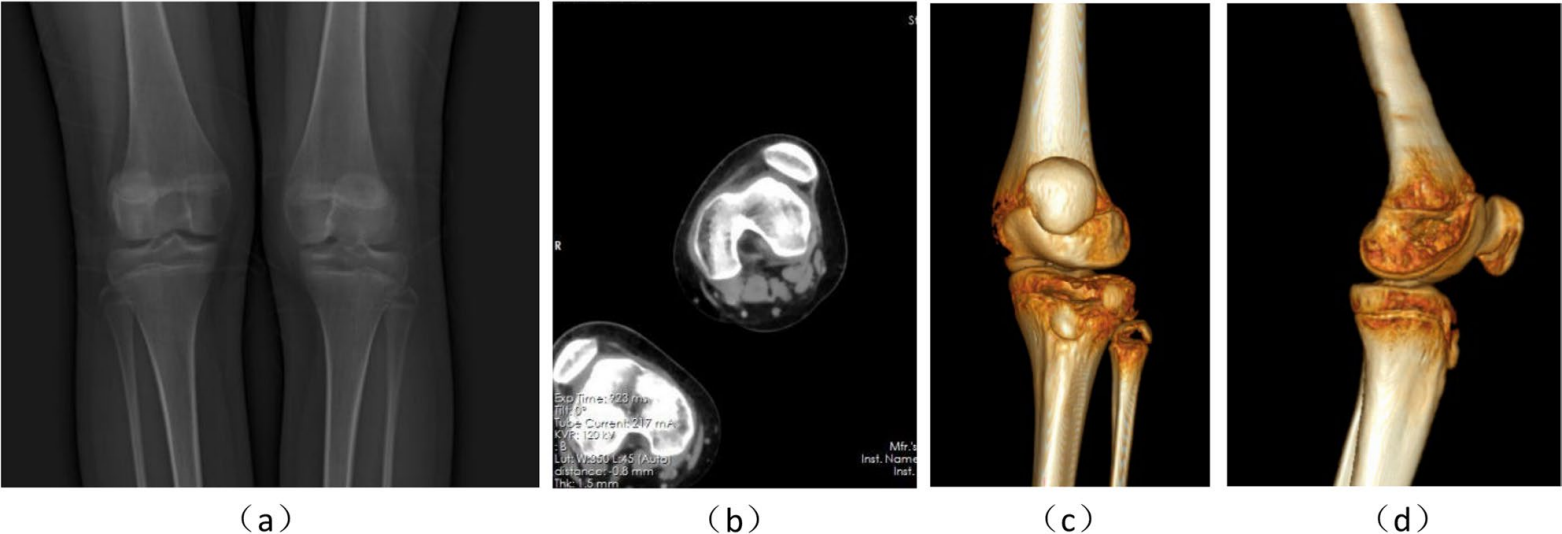
Preoperative radiograph and computed tomography (CT) images of the knee joint in case 3. **a** The anteroposterior image of both knee joints shows the unclosed epiphysis and malposition of the patellofemoral joint of both knee joints; **b** The horizontal CT image of the left knee joint shows complete lateral patellar prolapse; **c**, **d** CT three-dimensional reconstruction of the left knee joint shows obvious femoral internal rotation and tibial external rotation deformities that form a torsion deformity

An 11.6-year-old female had experienced a sluggish gait while walking since the age of 5 years. She often experienced weakness in both lower limbs when moving and was prone to falling. No special treatments were administered. The bilateral knee pain for > 1 month had no obvious cause, and the left knee joint disease was more obvious, as evidenced by squatting instability. Upon squatting, relying on the strength of the lower limbs, the patient could not stand on her own. Activities induced bilateral knee weakness and pain aggravation, and her daily life was significantly affected. Physical examination showed that the patella prolapsed outward and upward when the left knee joint was extended approximately 0° and returned to the normal position with approximately 90° of knee flexion. CT imaging of the left knee joint at 0° and 40° showed a femoral trochlear groove angle of 148.2°, Dejour type C trochlear dysplasia, trochlear dysplasia, patellofemoral malposition, a TT-TG measurement of 20.2 mm, and obvious tibial external rotation (Fig. 5). Kujala, IKDC, Lysholm, and VAS scores were 76.0, 64.4, 78.0, and 5.0, respectively. Due to the obvious tibial torsion deformity, severe symptoms, young age of the patient, and open epiphysis, the family of the patient considered that the risk of surgery was high and chose temporary conservative treatment without any surgical intervention. The patient’s family has opted to postpone surgical intervention until the patient reaches epiphyseal maturity.At the 12-month follow-up, the patient reported persistent symptoms of instability and limited squatting ability, with no improvement in the patellar dislocation frequency.

## Discussion

Epiphyseal closure is an important consideration when selecting a surgical method. In adults with habitual patellar dislocation with a closed epiphysis, trochlear dysplasia, insufficiency of the MPFL, patella alta, an abnormal Q angle, external rotation of the tibia, and genu valgum can aggravate patellar instability. Surgical options include lateral retinaculum release, MPFL reconstruction, trochleoplasty, tibial tuberosity displacement, and metaphyseal rotation osteotomy [6–11]. In children, E-HPD is caused by a combination of bone and soft tissue abnormalities. Common causes include trochlear dysplasia, patella alta, and tibial torsion deformity. When a patient attempts to straighten the knee joint from flexion, lateral dislocation occurs when the patella moves over the dysplastic lateral trochlea. The increased external rotation angle of the tibia may lead to an increase in the external rotational traction force at the end of the knee extension device, which is more obvious when the knee is straightened, resulting in lateral deviation of the patella. As the knee continues to flex, the patella returns to its normal position over the lateral trochlea. The epiphysis in children is not closed; therefore, bone surgery can damage the epiphysis and lead to premature closure. Soft-tissue surgery is often the only reasonable option for developing children. Some researchers recommend that surgical intervention for habitual patellar dislocation in children be deferred until epiphyseal closure, unless the condition significantly impairs the patient’s activities of daily living [12].

The three cases in this study were all children with severe E-HPD and patent epiphyses. They experienced weakness when squatting, and their daily lives were obviously affected. If they had not undergone early surgery, the bone and soft tissue deformities could have been further aggravated by growth and development, especially in the patella and trochlea. Early surgery can facilitate patellar and trochlear development and remodeling. Preventing the deterioration of symptoms, enabling the restoration of normal knee function, and reducing the incidence of knee pain, cartilage damage, and patellofemoral osteoarthritis in the future may also improve the mental health of pediatric patients.

Regarding the surgical treatment for pediatric E-HPD, Garin et al. [13] referred to surgical options for patellar dislocations in children. Among them, the Roux-Goldthwait procedure (lateral half-transposition of the patellar tendon) corrects lateral patellar displacement by adjusting the traction direction of the patellar tendon, whereas the Grammont procedure, a patellar ligament transfer-related technique, involves surgical intervention. Both techniques are used to improve patellar stability and are classic surgical procedures for children. Bicos et al. [14] investigated the biomechanics of the medial patellar retinaculum and found that the MPFL represented the major soft tissue structure restricting lateral dislocation or subluxation of the patella before entry into the femoral trochlea. Furthermore, it played an important dynamic stabilization role in the normal motion trajectory of the patellofemoral joint, representing 50–60% of the restriction force for the patella. Deie et al. also reported that MPFL reconstruction can effectively treat children with habitual patellar dislocations [15, 16] and that MPFL reconstruction alone was successful in treating four patients (six knees) with habitual flexion-type patellar dislocations without releasing lateral soft tissues. Furthermore, Yercan et al. [17] proposed an MPFL reconstruction technique using an autologous semitendinosus graft combined with adductor magnus tenodesis for children with bone immaturity and habitual patellar dislocations, with excellent outcomes in three children (four knees). Lateral retinaculum release was not performed in the above studies, most likely because the lateral retinaculum was not tense, and these cases were not E-HPD. In this study, extensive lateral soft tissue release improved the patellar trajectory after surgery and maintained the patella within the trochlear groove during knee extension and flexion, especially when partially adapted to the torsional deformity of the joint. Therefore, lateral retinaculum release is a significant part of the surgical intervention for habitual dislocation in children. The lateral release range of habitual patellar dislocations is wider than that of recurrent dislocations. The release range can be extended to approximately 2–3 cm above the lateral head of the quadriceps femoris, if necessary. Various soft tissues can be used for MFPL reconstruction, including the semitendinosus, vastus medialis, patellar tendon, adductor magnus muscle tendon, plantaris muscle tendon, and artificial ligaments [18, 19]. The technique of MPFL reconstruction by transposition of the adductor magnus tendon is suitable for children with an unclosed epiphysis, because drilling holes in the femoral condyle avoids the epiphysis. The two cases of MPFL reconstruction by transposition of the adductor magnus tendon in this study were children with immature patella alta. The tendons obtained intraoperatively achieved sufficient strength after weaving, and the postoperative prognosis was good, with no reported graft failures.

E-HPD is a rare and special type of patellar dislocation, with relatively few cases reported in the literature. Among the 672 patients with patellar dislocation treated at our research center in the past 10 years, three patients with E-HPD were recorded, all of whom were female children with an average age of 10.0 years and immature skeletal development. Different degrees of anatomical abnormalities, including trochlear dysplasia, knee torsion deformity, and patella alta, were observed on radiographs, CT scans, and magnetic resonance images. Diagnosis and treatment are difficult because improper treatment can seriously affect the knee joint function and even affect growth and development. Patients with minor dislocations and little functional impact can undergo conservative treatment by altering their movements and wearing patellar stabilization braces to reduce further injury to the patellofemoral joint. The gold standard for the surgical treatment of immature epiphyseal E-HPD remains to be established, and reports in the literature are scarce and controversial. MPFL reconstruction is the first-line treatment [8, 20]. However, the MPFL reconstruction alone lacks stability. In this study, the lateral retinaculum was released, in combination with MPFL reconstruction, to relieve the excessive lateral patellar traction caused by external rotation of the tibia, allow the patella to adapt to the trochlear trajectory formed by torsional deformity during knee extension and flexion, and ensure the stability of the entire knee joint. In this study, patellar dislocation could not be induced during passive knee extension and flexion in two patients under general anesthesia. The quadriceps femoris was speculated to require tension to induce patellar dislocation. Therefore, surgeons must be experienced and accurately judge the progress of the graft during MPFL reconstruction.

In this study, the combination of MFPL reconstruction and lateral retinacular release was used to treat E-HPD in two children with skeletal immaturity. In the short- and medium-term follow-ups, patellar stability and symptoms were significantly improved by MFPL reconstruction in combination with lateral soft tissue release, and the Kujala, IKDC, Lysholm, and VAS scores were significantly improved compared to preoperative values (Table 2). Both surgically treated patients resumed light sports activities (e.g., cycling) 3 months postoperatively and returned to full sports participation by 6 months, with no reports of instability during follow-up. Overall, the results are encouraging. Although a longer follow-up is required, these results indicate that MPFL reconstruction combined with lateral retinacular tissue release may be an effective option for treating pediatric patients with skeletally immature E-HPD. However, this surgical method has limitations for patients with severe knee torsion deformities, and the scope of its surgical indications requires exploration. Severe cases must be treated with bone surgery after attaining epiphyseal maturity.

**Table 2:** Comparison of preoperative and postoperative patient scores

Patient	Kujala score		IKDC score		Lysholm score		VAS score	
	Preoperative	Postoperative	Preoperative	Postoperative	Preoperative	Postoperative	Preoperative	Postoperative
1	79.0	91.0	70.1	87.4	70.0	86.0	3.0	1.0
2	84.0	94.0	66.7	80.5	71.0	90.0	5.0	2.0
3	76.0	-	64.4	-	78.0	-	5.0	-
Average score	79.7	92.5	67.1	84.0	73.0	88.0	4.3	1.5
Mean score (excluding non-operated patients)	81.5	92.5	68.4	84.0	70.5	88.0	4.0	1.5

In conclusion, pediatric E-HPD with epiphyseal immaturity is exceedingly rare, and the choice of treatment requires careful consideration. Surgical intervention must be performed with the utmost care to avoid epiphyseal injury. MFPL reconstruction and lateral retinacular release may be an effective treatment option for pediatric patients with E-HPD and skeletal immaturity.

## Data Availability

Data supporting the findings of this study are available from the corresponding author upon reasonable request.
